# The expression of ATP-sensitive potassium channels in human umbilical arteries with severe pre-eclampsia

**DOI:** 10.1038/s41598-021-87146-6

**Published:** 2021-04-12

**Authors:** Benlan Yin, Yujiao Zhang, Xiaohong Wei, Chunrong Pang, Ting Hou, Chao Yang, Yuzhi Ning, Xiaodong Fu

**Affiliations:** 1 Department of Obstetrics, The Affiliated Hospital of Southwest Medical University, No.8 Kangcheng Road, Luzhou, 646000 Sichuan Province China; 2 Department of Obstetrics, Guizhou Provincial People’s Hospital, No.83 Zhongshan East Road, Guiyang, 550000 Guizhou Province China

**Keywords:** Cardiovascular diseases, Immunological techniques

## Abstract

The aim of this study is to establish the expression of ATP-sensitive potassium channels(KATP) in human umbilical arteries with severe pre-eclampsia. Real-time quantitative PCR and western blotting were used to detect the mRNA and protein expression levels of KATP channel subunits Kir6.1 and SUR2B in human umbilical arteries from normal pregnant and those with severe pre-eclampsia, early onset severe pre-eclampsia and late onset severe pre-eclampsia. The mRNA and protein levels of SUR2B in the severe pre-eclampsia group were lower than those in the normal group (*P* < 0.001), and the expression of Kir6.1 was not statistically significant between the two groups (*P* > 0.05). The mRNA and protein levels of SUR2B in early onset severe pre-eclampsia group were lower than those in late onset severe pre-eclampsia group (*P* < 0.001). There was no significant difference in expression of Kir6.1 between the two groups (*P* > 0.05). The mRNA and protein expression levels of SUR2B in pregnant women with severe pre-eclampsia were lower than those in normal pregnant women, suggesting that the expression of the SUR2B of the KATP channel may be related to the occurrence and development of severe pre-eclampsia. Compared with late onset severe pre-eclampsia, the mRNA and protein expression levels of SUR2B were lower in the umbilical arteries of women with early onset severe pre-eclampsia, suggesting that the occurrence time of severe pre-eclampsia may be related to the extent reduced expression of the SUR2B of the KATP channel.

## Introduction

Severe pre-eclampsia (SP) is mainly characterized by hypertension, proteinuria and edema, including early onset (before 34 weeks of gestation) and late onset (after 34 weeks of gestation). The basic pathological changes are small vasospasm, ischemia and endothelial cell damage, and various organ dysfunctions may occur, which may be accompanied by fetal growth restriction, resulting in increased maternal and perinatal mortality. Therefore, it is necessary to strengthen the etiology of the disease and explore its mechanism of development to raise awareness of SP.

The ATP-sensitive potassium channel(KATP) is composed of the structural subunit inward rectifier potassium channel(Kir) and the regulating subunit sulfonylurea receptor(SUR), which are composed of Kir6.1, Kir6.2, SUR1, SUR2A, SUR2B^1–3^. It is widely expressed in a variety of excitable cells, and the subunits expressed in different tissues are not identical. Kir6.1 and SUR2B are more widely distributed in vascular smooth muscle cells^4^. KATP can control membrane potential, couple cell metabolism and activity, regulate smooth muscle cell proliferation and apoptosis, modulate vascular remodeling and play an important role in controlling function of vascular smooth muscle^5,6^.

Previous ion channel studies of the chorionic plate arteries suggested a correlation between severe pre-eclampsia and ion channel gene expression^7^. As a major potassium channel, the role of KATP in the development of severe pre-eclampsia requires further study. The umbilical artery is an important hub connecting the fetus and the mother. Pregnant women with severe pre-eclampsia often experience intrauterine growth restriction or intrauterine distress. Therefore, this experiment aims to detect and compare the mRNA and protein expression levels of the KATP channel subunits Kir6.1 and SUR2B in normal and severe pre-eclampsia umbilical arteries by real-time quantitative PCR (QPCR) and western blotting. On this basis, the mRNA and protein expression levels of KATP subunits in human umbilical arteries in late onset severe pre-eclampsia and early onset severe pre-eclampsia were detected to explore the correlation between changes in this channel and severe pre-eclampsia. From the molecular level to the mechanism of severe pre-eclampsia, the results of this study may provide a basis for the further study of severe pre-eclampsia at the genetic level.

## Results

### Basic situation of the research objects

All pregnant women had a clear gestational age. There was no significant difference in age between the 40 normal pregnant women and 40 severe pre-eclampsia pregnant women (*P* > 0.05). There was no significant difference in gestational age (*P* > 0.05), but the blood pressure difference was statistically significant (*P* < 0.001). See Table 1 for details. There was no significant difference in age between the 20 pregnant women with late onset severe pre-eclampsia and the 20 pregnant women with early onset severe pre-eclampsia (*P* > 0.05). There was no significant difference in blood pressure between the two groups (*P* > 0.05). The difference in gestational age was statistically significant (*P* < 0.001), see Table 2.

**Table 1:** The basic information of normal pregnancy patients and severe pre-eclampsia patients.

Parameter	NP group	SP group	*P*-value
Age	28.03 ± 3.98	29. 08 ± 5.29	0.319
Gestational age	38.98 ± 0.96	38.50 ± 1.20	0.053
Blood pressure	117.50 ± 7.13/74.65 ± 6.54	169.88 ± 10.41/108.70 ± 10.74	*P* < 0.001/*P* < 0.001

**Table 2:** The basic information of late onset severe pre-eclampsia patients and early onset severe pre-eclampsia patients.

Parameter	LSP group	ESP group	*P*-value
Age	29.40 ± 6.43	28. 15 ± 4.99	0.496
Gestational age	38.06 ± 1.64	32.20 ± 1.32	*P* < 0.001
Blood pressure	167.05 ± 11.10/106.70 ± 10.04	175.85 ± 17.08/111.20 ± 13.82	0.061/0.246

### Comparative analysis of the normal group and the severe pre-eclampsia group

**Figure 1:**
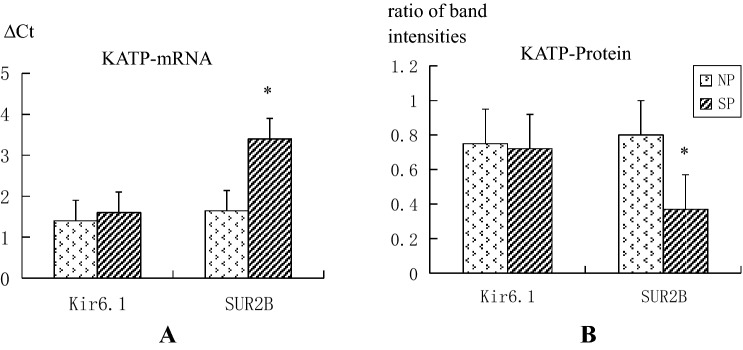
Bar graph of the mRNA and protein expression levels of Kir6.1 and SUR2B in normal patients and severe pre-eclampsia patients.

The QPCR results of Kir6.1 and SUR2B subunits in umbilical arteries from the normal group and the severe pre-eclampsia group are shown in Table 3 and Fig. 1A. There was no significant difference in the expression of Kir6.1 subunit between the two groups (*P* = 0.234). The expression of SUR2B subunit was significantly different (*P* < 0.001). The relative expressionvalue was calculated to be 0.5013 ± 0.1809, suggesting that SUR2B subunit mRNA expression is lower in umbilical arteries of the severe pre-eclampsia group than in the normal group.

**Table 3:** The mRNA expression levels of Kir6.1 and SUR2B in normal patients and severe pre-eclampsia patients.

Parameter	n	NP group ΔCt	SP group ΔCt	*P*-value
Kir6.1	40	1.4297 ± 0.5665	1.6016 ± 0.7089	0.234
SUR2B	40	1.6435 ± 0.6663	3.4066 ± 0.5452	*P* < 0.001

**Figure 2:**
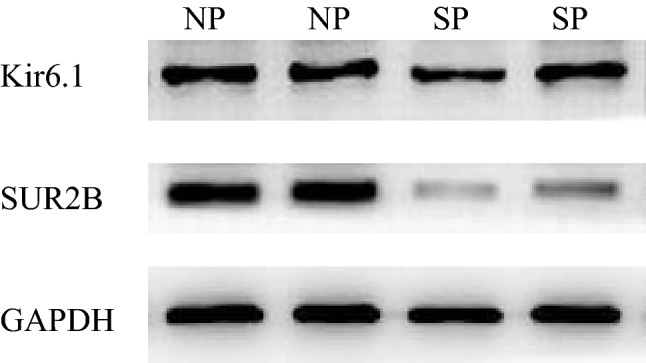
Imaging of the protein expression levels of Kir6.1 and SUR2B in normal patients and severe pre-eclampsia patients.

Densitometry analysis of the western blot results of the Kir6.1 and SUR2B subunits in the two groups is shown in Table 4 and Figs. 1B and 2. The Kir6.1/GAPHD values were not significantly different between the two groups (*P* = 0.367); however, the SUR2B/GAPHD values were extremely different (*P* < 0.001). The SUR2B subunit protein level was lower in umbilical arteries of the severe pre-eclampsia group than the normal group.

**Table 4:** The protein expression levels of Kir6.1 and SUR2B in normal patients and severe pre-eclampsia patients.

Parameter	n	NP group	SP group	*P*-value
Kir6.1	40	0.7484 ± 0.1130	0.7222 ± 0.1428	0.367
SUR2B	40	0.8077 ± 0.1241	0.3701 ± 0.1209	*P* < 0.001

### Comparative analysis of umbilical arteries in patients with late onset severe pre-eclampsia and early onset severe pre-eclampsia

**Figure 3:**
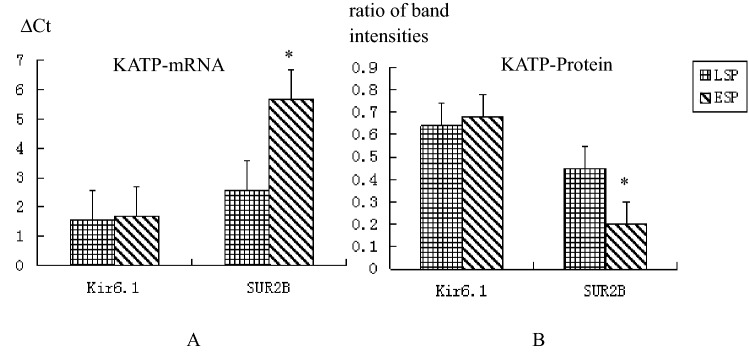
Bar graph of the mRNA and protein expression levels of Kir6.1 and SUR2B in late onset severe pre-eclampsia patients and early onset severe pre-eclampsia patients.

The QPCR results of the LSP group and the ESP group are shown in Table 5 and Fig. 3A. There was no significant difference in the ΔCt values of the Kir6.1 subunit between the LSP and ESP groups (*P* = 0.333). There was a statistically significant difference in the ΔCt values of the SUR2B subunit between two groups (*P* < 0.001). The relative expressionvalue was calculated to be 0.1176 ± 0.0040, suggesting that the SUR2B subunit mRNA level was lower in umbilical arteries of the ESP group than in LSP group.

**Table 5:** The mRNA expression levels of Kir6.1 and SUR2B in late onset severe pre-eclampsia patients and early onset severe pre-eclampsia patients.

Parameter	n	LSP group ΔCt	ESP group ΔCt	*P*-value
Kir6.1	20	1.5402 ± 0.5072	1.6921 ± 0.4710	0.333
SUR2B	20	2.5685 ± 0.5705	5.6608 ± 0.5244	*P* < 0.001

**Figure 4:**
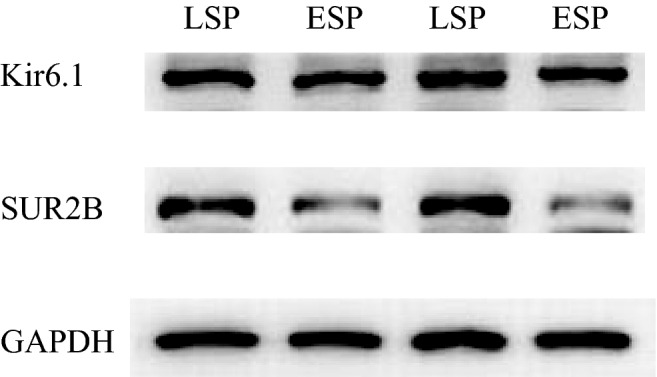
Imaging of the protein expression levels of Kir6.1 and SUR2B in late onset severe pre-eclampsia patients and early onset severe pre-eclampsia patients.

Densitometry analysis of the western blot results of the LSP group and the ESP group are shown in Table 6 and Figs. 3B and 4.There was no significant difference in Kir6.1/GAPHD values between the two groups (*P* = 0.159). The SUR2B/GAPHD values were significantly different between the two groups (*P* < 0.001). The expression level of SUR2B subunit protein was lower in umbilical arteries of the ESP group than in LSP group.

**Table 6:** The protein expression levels of Kir6.1 and SUR2B in late onset severe pre-eclampsia patients and early onset severe pre-eclampsia patients.

Parameter	n	LSP group	ESP group	*P*-value
Kir6.1	20	0.6436 ± 0.0799	0.6874 ± 0.1106	0.159
SUR2B	20	0.4544 ± 0.0997	0.2047 ± 0.0344	*P* < 0.001

## Discussion

First, regarding the role of KATP in vascular-related diseases, studies have shown that the opening of KATP is an endogenous protective mechanism against ischemia, hypoxia, cardiac hypertrophy, etc. Moreover, KATP on the cell membrane and KATP on the mitochondrial membrane can protect against myocardial ischemia–reperfusion injury through different mechanisms^8^. In response to this finding, a large number of studies have been conducted on KATP channel function in recent years, and it has been shown that KATP can play a cardioprotective role in many species, which are widely used to study myocardial ischemia and cardiac hypertrophy^9^. Another study demonstrated that compared with rats with normal blood pressure, the expression levels of Kir6.1 subunit and SUR2B subunit in the aortic vascular smooth muscle cells of hypertensive rats were reduced^10^. In the clinical treatment of hypertension, the main mechanism of action of KATP is to promote cell membrane superclosure to prevent L-type Ca^2+^ influx, inhibit the storage of Ca^2+^ in cells, reduce the amount of intracellular Ca^2+^, relax the smooth muscle, and thus protect against hypertension-induced injury^11^. When hypoxic pulmonary hypertension occurs, pulmonary vascular endothelial cells synthesize and secrete vasoactive substances such as endothelin-1 (ET-1) and nitric oxide (NO), which act on their corresponding receptors to induce intracellular signal transduction acting on the KATP channel of pulmonary artery smooth muscle cell membrane. This causes contraction or relaxation of pulmonary artery smooth muscle cells^12^. There was no significant difference in the mRNA expression of Kir6.1 subunit between the pulmonary hypertension model group and the normal group. It is believed that the occurrence of pulmonary hypertension may be associated with inhibition of KATP channels and reduction of SUR2B subunits.

Second, regarding the expression and significance of potassium channels in severe pre-eclampsia, QPCR and western blotting experiment were used to confirm that down-regulated Kv7 channel function in tension regulation of chorionic plate arteries in women with pre-eclampsia could be associated with considerably altered expression profiles of Kv7 subunits^7^. Studies have shown that the regulation of K + channels is an important mechanism in the adaptation of uterine vascular tone to pregnancy^13^. Our previous experiments showed that, compared to normal pregnant women, there was no significant change in the expression of large-conductance calcium-activated potassium channel (BKCa) α subunit mRNA or protein in the uterine arterioles and mesenteric arteries of patients with severe pre-eclampsia as determined by QPCR and western blotting, respectively. The mRNA and protein expression levels of the β1 subunit were significantly decreased, further suggesting that functional changes in the ion channel BKCa may be involved in the etiology of severe pre-eclampsia.

In the present study, QPCR and western blot results showed that compared to normal pregnant women, the expression of the SUR2B subunit of the KATP channel was significantly reduced in the umbilical arterial smooth muscle cells of women with severe pre-eclampsia, but the expression of the Kir6.1 subunit was not statistically significant. Since the Kir6.1 subunit is a structural subunit and the SUR2B subunit is a functional subunit, our results suggest that reduced activity of the SUR2B subunit of KATP channel is involved in the development of severe pre-eclampsia. Abnormal expression of the SUR2B subunit may be one of the factors leading to systolic dysfunction in umbilical arteries. In combination with studies on other vascular diseases, it can be inferred that KATP agonists could improve the function of vascular artery in severe pre-eclampsia and cause vasodilation, thereby improving fetal blood supply.

Severe pre-eclampsia includes early onset (before 34 weeks of gestation) and late onset (after 34 weeks of gestation). Studies on early and late onset severe pre-eclampsia mainly focus on the comparative analysis of clinical indicators and related gene expression. For example, some scholars have reported that ABI3BP, C7, HLA-G and IL2RB might contribute to the development of early form of severe pre-eclampsia^14^. There are few reports comparing potassium channel expression in patients with early onset and late onset severe pre-eclampsia.

In the present study, the expression levels of the KATP channel subunits Kir6.1 and SUR2B in the umbilical arteries of patients with early onset severe pre-eclampsia and late onset severe pre-eclampsia were compared by QPCR and western blotting. The results showed that there were no significant differences in the mRNA and protein expression of Kir6.1 subunit between the two groups, while mRNA and protein expression of SUR2B subunit was lower in early onset severe pre-eclampsia. These data suggest that functional loss of KATP channel subunit SUR2B is more serious in early onset severe pre-eclampsia than in patients with late onset severe pre-eclampsia. Therefore, we hypothesize that early versus late occurrence of severe pre-eclampsia may be related to the degree of KATP channel SUR2B subunit loss.

## Conclusions

The decrease in KATP channel SUR2B subunit expression may be related to the occurrence and development of severe pre-eclampsia; however, this study did not address we its causal relationship. One possibility is that the reduction of SUR2B causes the potassium channel to be inhibited, which leads to vasoconstriction occurs, ultimately resulting in severe pre-eclampsia. Alternately, the occurrence of severe pre-eclampsia leads to changes in the SUR2B subunit of the KATP channel, or the interaction between SUR2B expression and pre-eclampsia causes persistent damage, ultimately leading to a reduction in SUR2B subunit expression and the development of severe pre-eclampsia. Such hypotheses need to be further studied at the genetic level.

KATP channel agonists such as iptakalim, pinacidil and nicorandil are not currently used for the clinical treatment of severe pre-eclampsia. In the future, we can develop related targeted drugs and conduct animal experiments or clinical randomized double-blind experiments with the ultimate aim of controlling the occurrence and development of severe pre-eclampsia and reducing the harm of this disease to maternal and neonatal health.

## Materials and methods

### Research object and experimental materials

Pregnant women with severe pre-eclampsia and normal pregnant women admitted to the Obstetrics Department of the Affiliated Hospital of Southwest Medical University at the same time were selected as the experimental subjects. The diagnostic criteria were based on current Chinese 9th edition of Obstetrics and Gynecology (edited by Xie Xing)^15^: pre-eclampsia accompanied by one of the following conditions: systolic blood pressure at ≥ 140 mmHg or diastolic blood pressure at ≥ 90 mmHg; platelet count < 100 000/μL; transaminases more than twice the normal value; creatinine ≥ 1.1 mg/dL; pulmonary edema; new neurological abnormalities. The exclusion criteria included the following: multiple pregnancy; single umbilical artery; comorbidity with endoscopic disease or infectious disease; comorbidity with other pregnancy-specific diseases or obstetric complications including gestational diabetes, intrahepatic cholestasis of pregnancy, placenta previa, placental abruption, etc.; and history of smoking or alcohol abuse. The experimental materials were umbilical artery specimens collected during vaginal delivery or cesarean section from pregnant women who meet the inclusion criteria. The sample size of the study was as follows: a total of 120 cases including 40 cases of normal pregnant (NP) women of the childbearing age and 40 cases of severe pre-eclampsia (SP), 20 cases of late onset severe pre-eclampsia (LSP) and 20 cases of early onset severe pre-eclampsia (ESP). This study was approved by the hospital’s ethics committee. All pregnant women were informed about the postpartum specimens and provided written informed consent.

### Specimen collection, separation and cryopreservation

When a placenta meeting the inclusion criteria was delivered, an umbilical cord specimen with a length of about 5 cm was taken immediately (following aseptic procedures) and transferred to the laboratory. The umbilical cord was submerged in the pre-cooled enzyme-free water and separated on ice. The umbilical artery was placed in an enzyme-free cryotube and rapidly transferred to a − 80 °C ultra-low temperature freezer for storage prior to QPCR and western blotting.

### QPCR experiment

**Figure 5:**
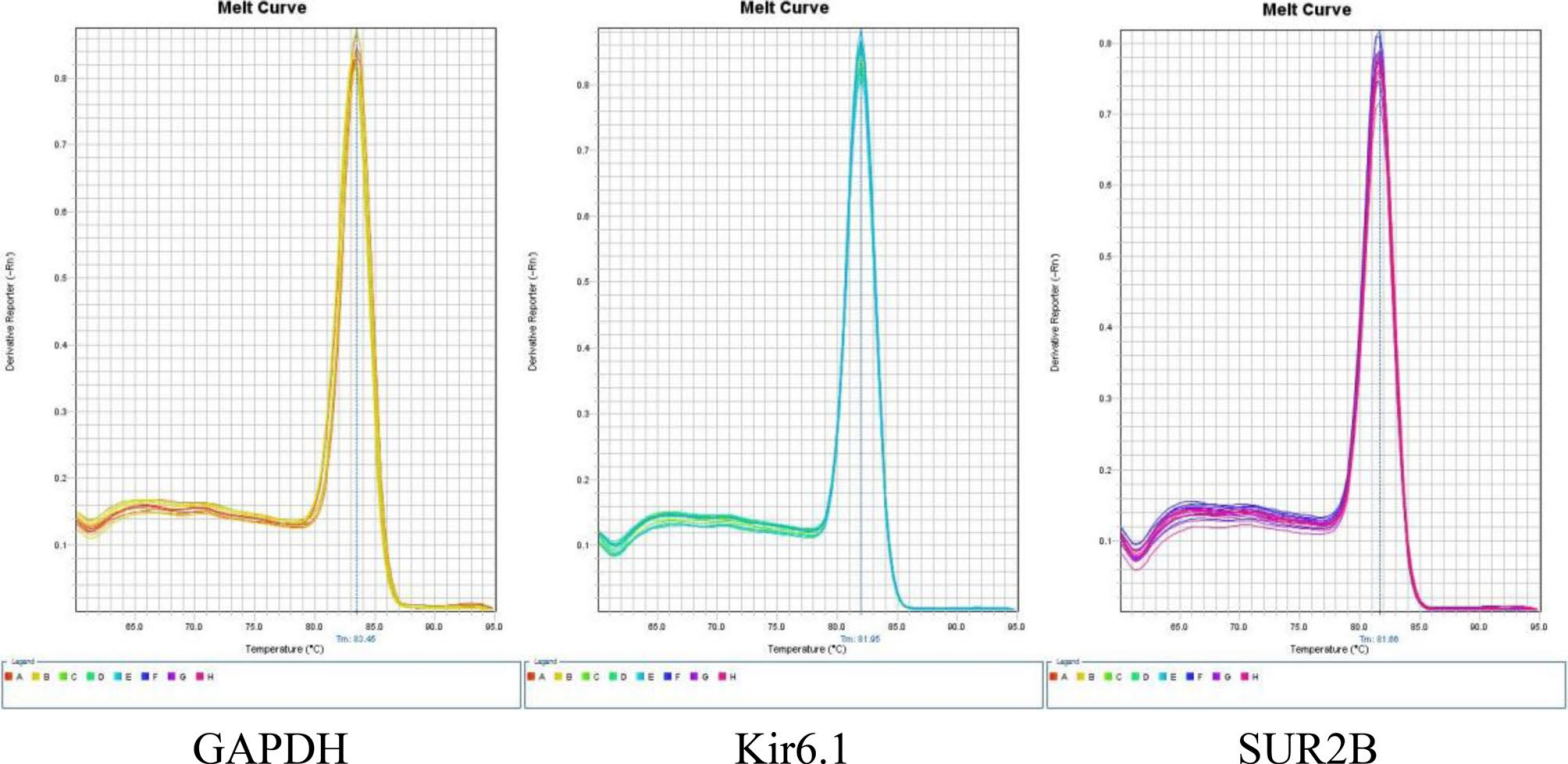
The melting curves of GAPDH, Kir6.1 subunit and SUR2B subunit.

Total RNA extraction from umbilical arteries was performed according to the instructions of a Tiangen Total RNA Extraction Kit (Tiangen Biotech, Beijing, China). The tissue was repeatedly ground to a fine powder form. Poured into liquid nitrogen continuously to avoid the increase of tissue temperature and degrading RNA. After grinding, quickly took about 80 mg of sample powder, added to 1 ml lysis buffer. Sufficiently lysed the mixture, added 200 μL chloroform, and centrifuged the mixture at 12,000 rpm for 10 min. Then collected 400μL supernatant , added 200 μL anhydrous ethanol , and transferred the mixture to an adsorption column, washed with 500 μL deproteinized solution, rinsed and dried, and then dissolved in 30μL enzyme-free water. The extracted RNA concentration and purity were measured using a nucleic acid concentration detector (Thermo Scientific, Waltham, MA, USA). Reverse transcriptase (Toyobo, Osaka, Japan) was used to reverse-transcribe total RNA into complementary DNA(cDNA), and QPCR was performed according to the instructions of a QuantiNova SYBR Green PCR Kit (QIAGEN, Hilden, Germany) to obtain a corresponding Ct value. The upstream and downstream primers of KIR6.1, SUR2B and GAPDH genes were designed and synthesized by Sangon Biotech (Shanghai, China). Each primer sequence is shown in Table 7. In Fig. 5, the melting curves of GAPDH, Kir6.1 subunit and SUR2B subunit are all single peaks, narrow and sharp, indicating that the designed primers are acceptable and no dimers are produced.

**Table 7:** Forward and reverse primer sequences.

Gene	Primer sequence	Tm (°C)	PCR product size (bp)
GAPDH F	5′-CCACTCCTCCACCTTTG-3’	52.7	106
GAPDH R	5′-CACCACCCTGTTGCTGT-3’	55.7	106
KIR6.1 F	5′-GATCATCTGCCACGTGATTGA-3’	58.0	150
KIR6.1 R	5′-GCAATGTAGGAGGTTCGTGCT-3’	60.0	150
SUR2B F	5′-CGGGACATAACCTGAGATGG-3’	59.8	130
SUR2B R	5′-ATCACGGCTGGCATAAAGAG-3’	57.8	130

Tm: DNA melting temperature.

The QPCR results were the Ct value of each gene in each sample, and the ΔCt value was calculated as the Ct value of the target gene minus the Ct value of the reference gene. The larger the ΔCt value, the lower the substrate concentration. The ΔΔCt value was calculated as the experimental group gene ΔCt value minus the control group ΔCt value, and the 2^-ΔΔCt^ (relative expression) value indicated that the experimental group gene was a multiple of the control group gene.

### Western blotting experiment

Tissue samples were ground as described above, after grinding, quickly took about 80 mg of sample powder, then added to 800 μL lysis buffer and protease inhibitor (protease inhibitor/lysis buffer: 1:100). Samples were fully lysed and centrifuged at 12,000 rpm for 15 min. The supernatant was then collected and subjected to sodium dodecyl sulfate polyacrylamide gel electrophoresis (SDS-PAGE). The percentage of separating gel used for WB was 10% while the concentrated gel was 5%. Loaded 40ug protein into each well. Proteins were then transferred to PVDF membranes, blocked in 5% skim milk at room temperature for 2 h, and then incubated in primary antibody at 4 °C overnight. Primary antibodies used include glyceraldehyde phosphate dehydrogenase(GAPDH) (Bioworld, Irving, TX, USA) 1:10,000, Kir6.1 (Alomone, Jerusalem, Israel) 1:400, SUR2B (Abcam, Cambridge, UK) 1:500. Phosphate buffered saline with Tween 20 (PBST, Tween 20/PBS: 1:1000) was used to wash PVDF membranes, which were incubated in secondary antibody for 2 h at room temperature. Secondary antibodies used include Goat anti-Mouse IgG (H&L)-HRP (GAPDH secondary antibody, Bioworld, Irving, TX, USA) 1:10,000, Goat anti-Rabbit IgG Secondary Antibody (Kir6.1 and SUR2B secondary antibody, Millipore, Burlington, MA, USA) 1:10,000. The PVDF membrane again washed with PBST, then imaged by a chemiluminescence imaging system (Fusion Solo, Paris, France).

Densitometry analysis of the images obtained by western blotting was performed using Quantity-One (Bio-Rad, California, USA). The relative expression levels of the target proteins Kir6.1 and SUR2B were obtained as the ratio of band intensities to those of the internal reference protein GAPDH.

### Statistical analysis of experimental results

Data were compared via Student’s t test using SPSS version 19.0 (IBM Corporation, Aramonk, NY, USA). The results are expressed as the mean ± standard deviation (± s). If *P* < 0.05, then the difference was considered statistically significant.

### Ethics approval and consent to participate

This study protocol was approved by The Ethics Committee of The Affiliated Hospital of Southwest Medical University. This study was performed according to the principles expressed in the Declaration of Helsinki and informed consent was obtained from the subjects.

## Supplementary Information

Supplementary Figures

## Data Availability

The datasets that support the findings of this study are available from the corresponding author, Xiaodong Fu, upon reasonable request.

## Abbreviations

KATP: ATP-sensitive potassium channel
SP: Severe pre-eclampsia
Kir: Inwardly rectifying potassium channel
SUR: Sulphonylurea receptor
QPCR: Real-time fluorescent quantitative PCR experiment
NP: Normal pregnant
LSP: Late onset severe pre-eclampsia
ESP: Early onset severe pre-eclampsia
cDNA: Complementary DNA
SDS-PAGE: Sodium dodecyl sulfate polyacrylamide gel electrophoresis
GAPDH: Glyceraldehyde phosphate dehydrogenase
PBST: Phosphate buffered saline with Tween 20
ET-1: Endothelin-1
NO: Nitric oxide
BKca: Large-conductance calcium-activated potassium channels
