# Supplementary material for: The expression of ATP-sensitive potassium channels in human umbilical arteries with severe pre-eclampsia
Source: Sci Rep. 2021 Apr 12;11:7955. doi: 10.1038/s41598-021-87146-6 (PMC8041753; doi:10.1038/s41598-021-87146-6)
Supplement: Supplementary file 1 — Supplementary Figures [file 41598_2021_87146_MOESM1_ESM.docx]

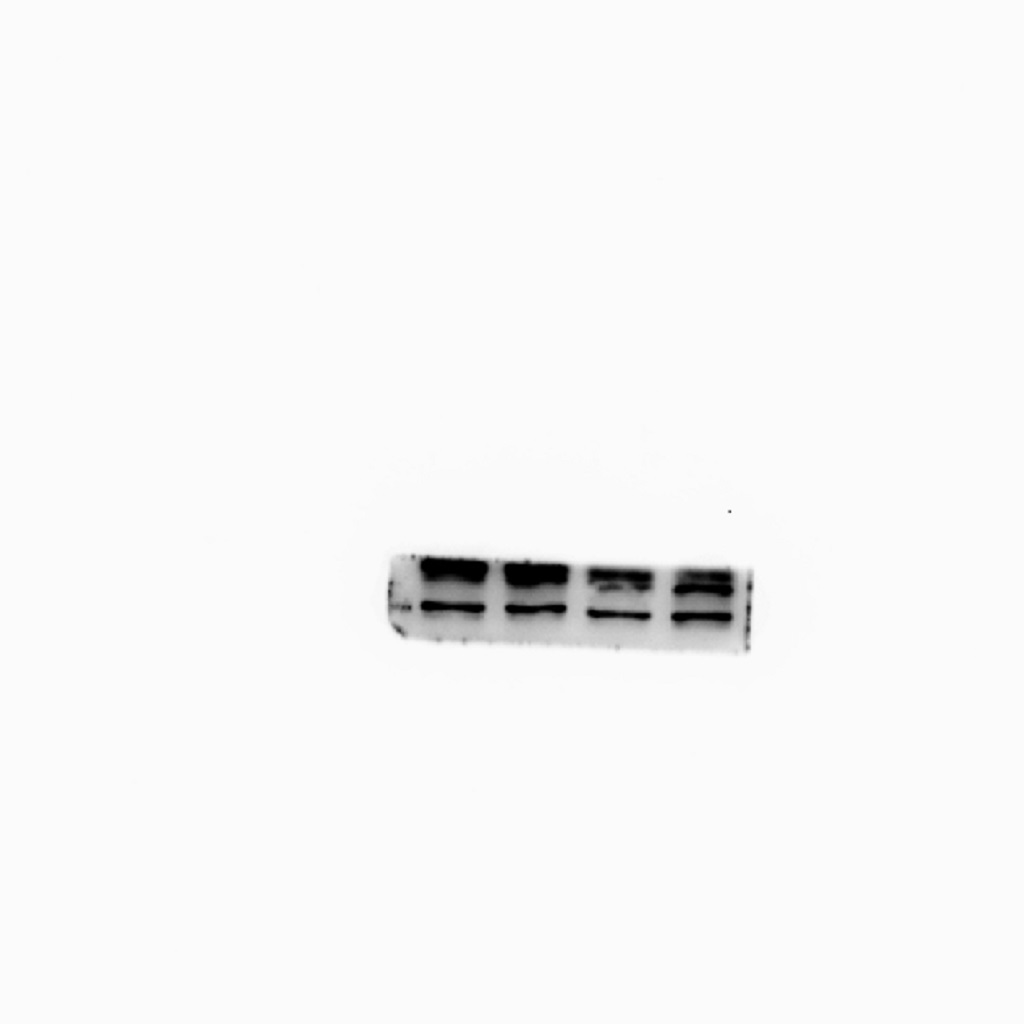


Figure 1 Imaging of the protein expression levels of Kir6.1 in normal patients and severe pre-eclampsia patients. The primary antibody of Kir6.1 (Alomone, Jerusalem, Israel) is polyclonal antibody. Predicted molecular weight: 60 kDa.


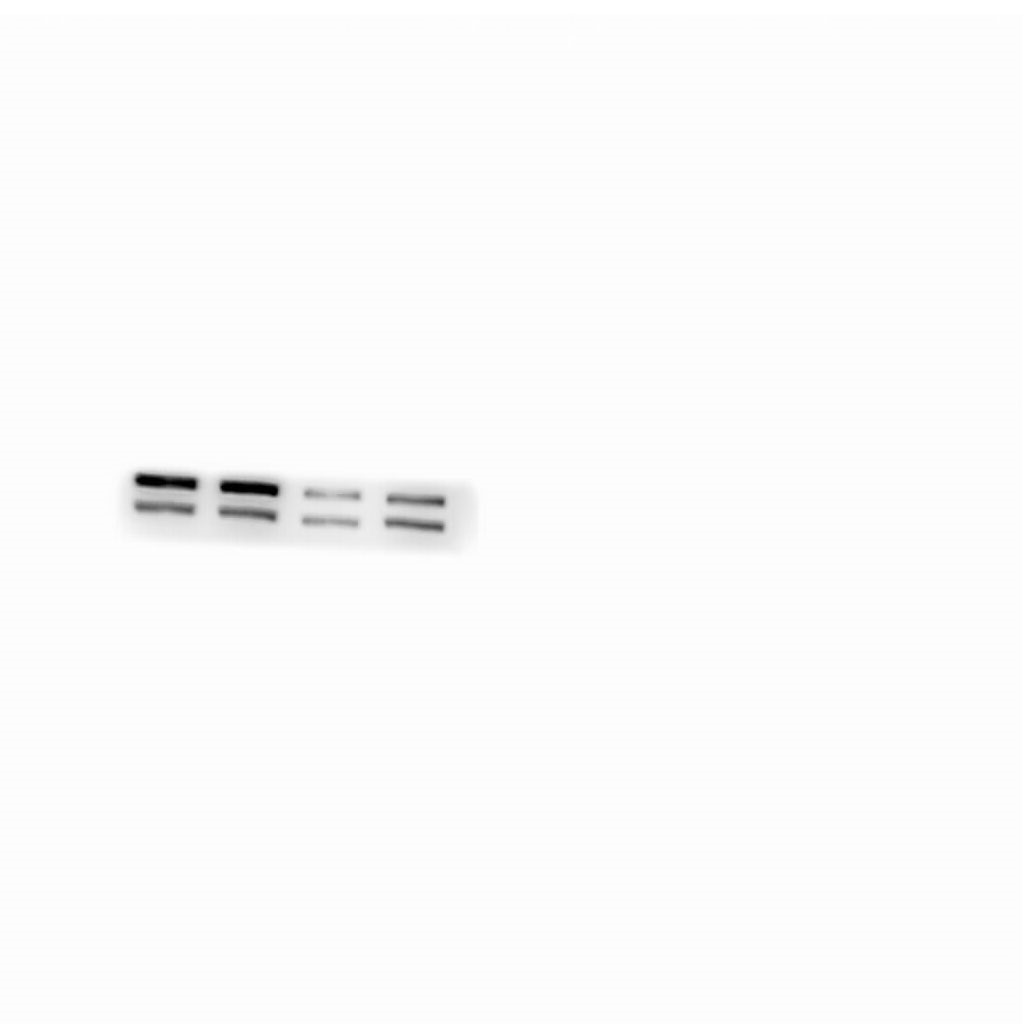


Figure 2 Imaging of the protein expression levels of SUR2B in normal patients and severe pre-eclampsia patients. The primary antibody of SUR2B (Abcam, Cambridge, UK) is polyclonal antibody. Predicted molecular weight: 174 kDa.


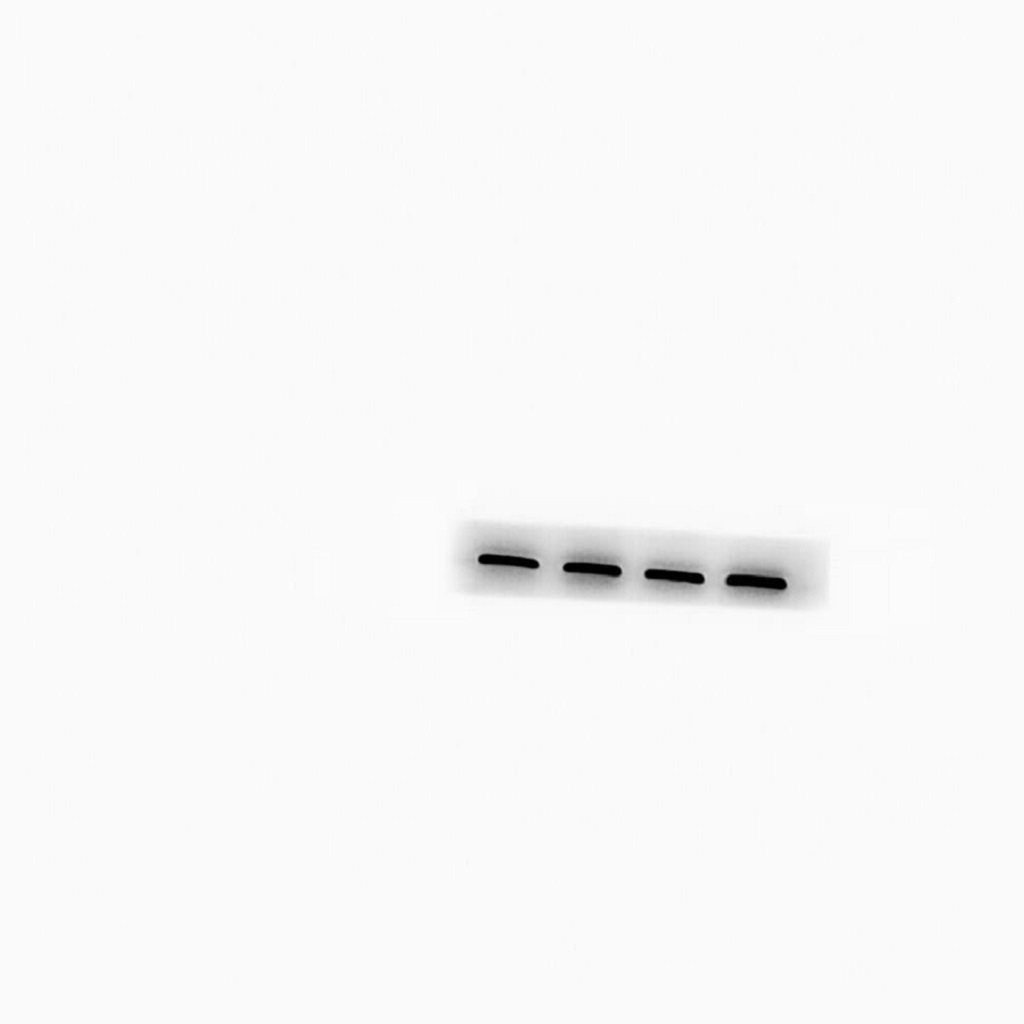


Figure 3 Imaging of the protein expression levels of GAPDH in normal patients and severe pre-eclampsia patients. The primary antibody of GAPDH (Bioworld, Irving, TX, USA) is monoclonal antibody. Predicted molecular weight: 36 kDa.


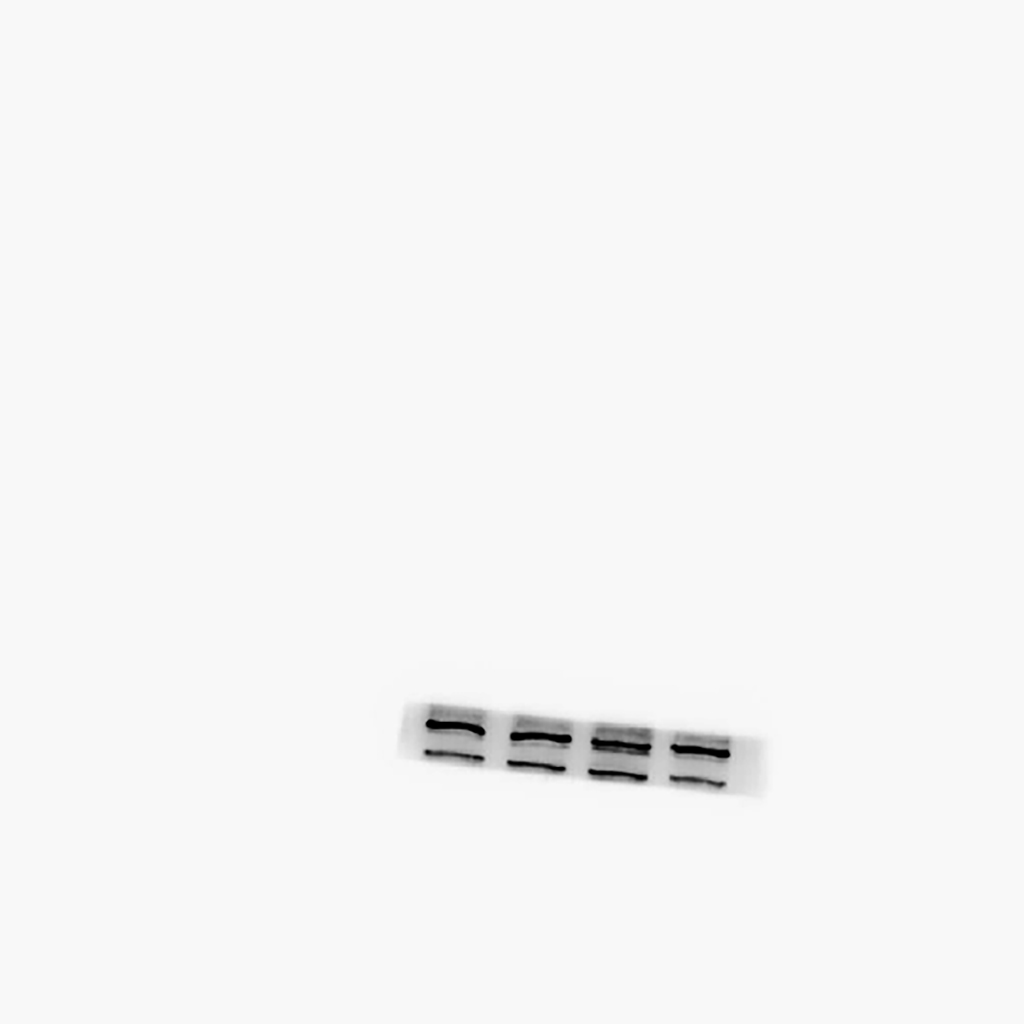


Figure 4 Imaging of the protein expression levels of Kir6.1 in late onset severe pre-eclampsia patients and early onset severe pre-eclampsia patients. The primary antibody of Kir6.1 (Alomone, Jerusalem, Israel) is polyclonal antibody. Predicted molecular weight: 60 kDa.


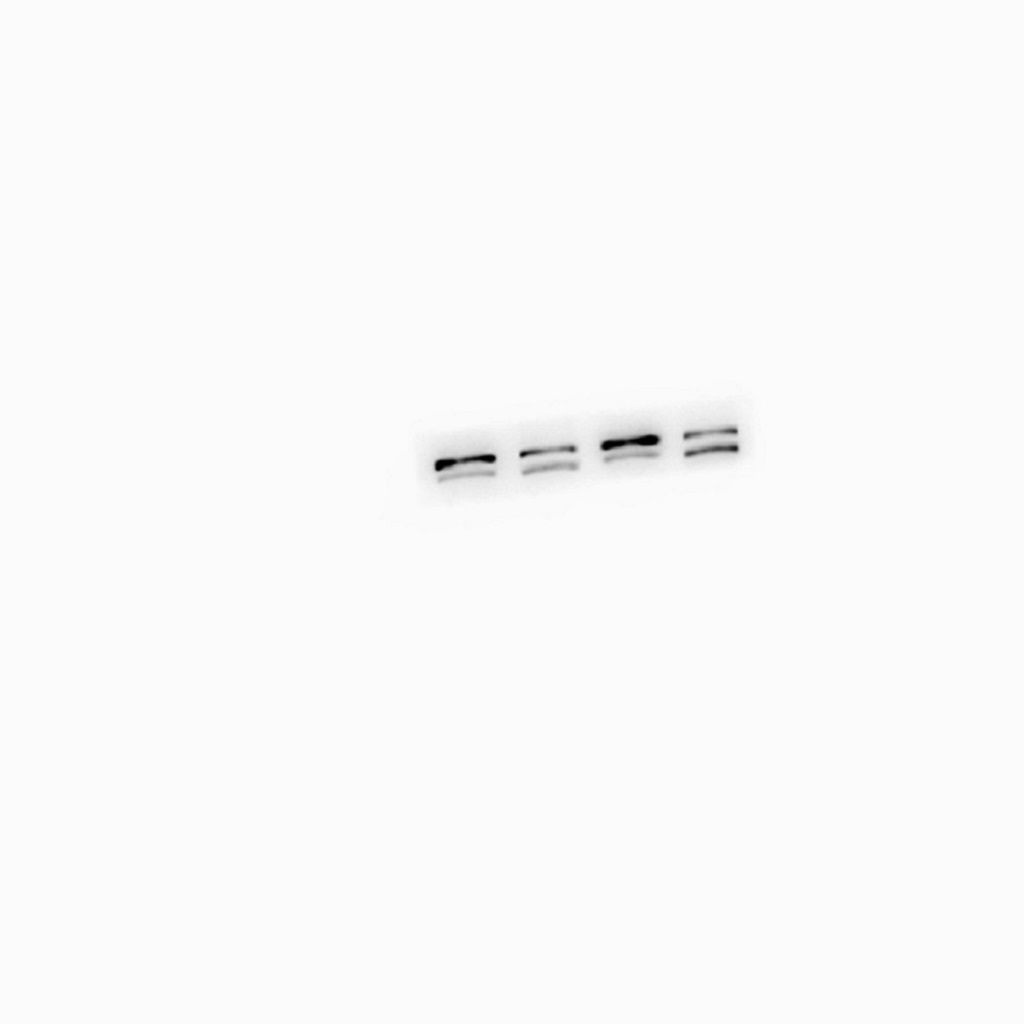


Figure 5 Imaging of the protein expression levels of SUR2B in late onset severe pre-eclampsia patients and early onset severe pre-eclampsia patients. The primary antibody of SUR2B (Abcam, Cambridge, UK) is polyclonal antibody. Predicted molecular weight: 174 kDa.


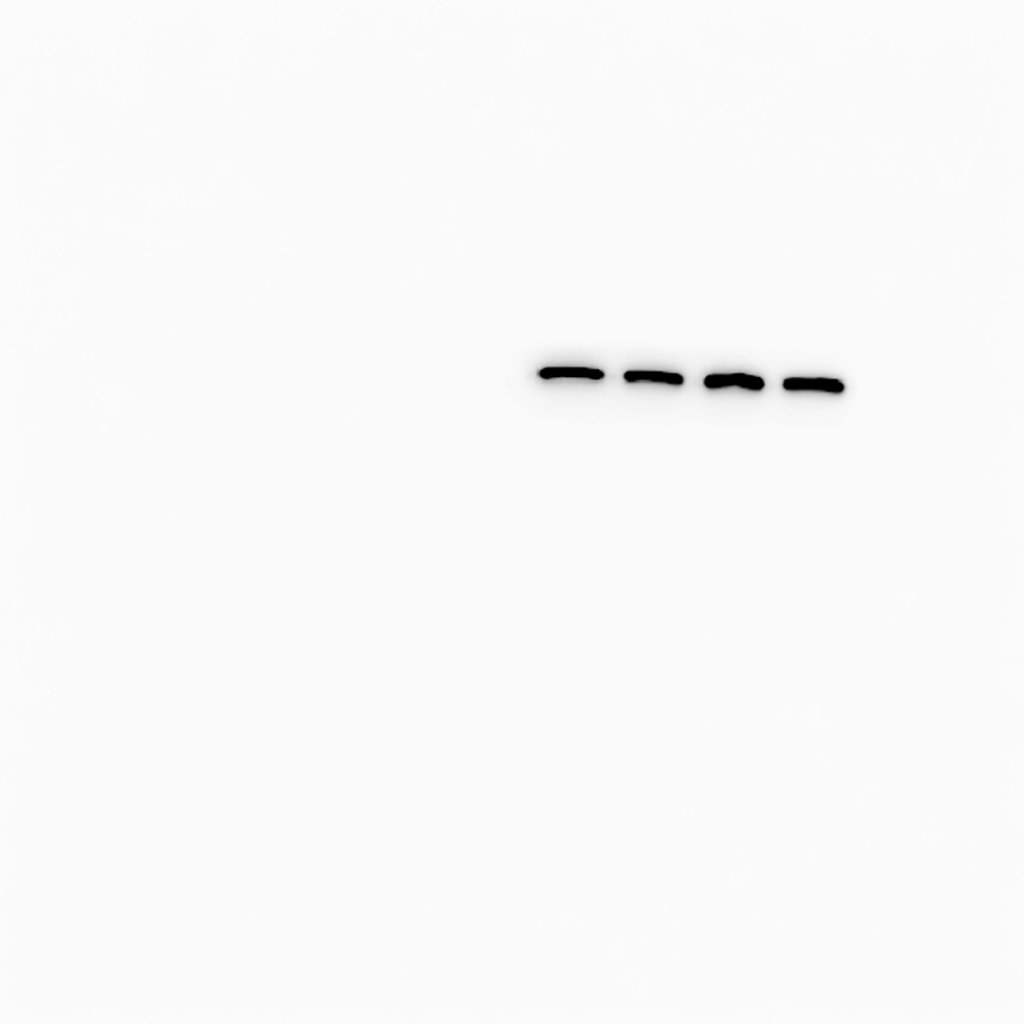


Figure 6 Imaging of the protein expression levels of GAPDH in late onset severe pre-eclampsia patients and early onset severe pre-eclampsia patients. The primary antibody of GAPDH (Bioworld, Irving, TX, USA) is monoclonal antibody. Predicted molecular weight: 36 kDa.
